# Natural immunomodulator preimplantation factor PIF affected cancer growth in malignant melanomas

**DOI:** 10.1186/1479-5876-12-S1-P13

**Published:** 2014-05-06

**Authors:** Beatrix Kotlan, Gyorgy Naszados, József Továri, János F László, Maria Godeny, Eytan R Barnea

**Affiliations:** 1 Department of Molecular Immunology and Toxicology, National Institute of Oncology, Budapest, Hungary; 2 Center of Surgical and Molecular Tumorpathology; National Institute of Oncology, Budapest, Hungary; 3 Department of Radiological Diagnostics, National Institute of Oncology, Budapest, Hungary; 4 Department of Experimental Pharmacology, National Institute of Oncology, Budapest, Hungary; 5 Department of Tumorbiology, National Koranyi Institute of TB and Pulmonology, Budapest, Hungary; 6 Department of Computer Science, Faculty of Informatics, University of Debrecen, Debrecen, Hungary; 7 SIEP, Society of the Investigation of Early Pregnancy, Cherry Hill, New Jersey, USA; 8 BioIncept LLC, Cherry Hill, USA; 9 UMDNJ University of Medicine and Dentistry of New Jersey – Robert Wood Johnson Medical School, New Jersey, USA

## Background and objectives

Malignant and trophoblastic cells share common features in terms of migration and invasion, while they represent striking differences also (1). Our results on immune mechanisms in pregnancy failure (2) and use of immunotherapeutics (3) fostered the present new approach: Pregnancy derived compounds for potential growth controlling effect in metastatic melanomas were studied.

## Methods

Preimplantation factor (PIF), a novel peptide secreted by viable embryos was selected (4), as its immune regulatory effects were advantageous (5). Based on our tumorimmunological project (Ethical permission ETT TUKEB 1642-02/2010), minor tissue samples from surgically removed lymphnodes of patients with metastatic melanomas were processed. Primary cultures were set up in special conditions with or without immunomodulatory PIF. In a parallel system, we transfused human HT199 melanoma cells into immunodeficient NOD.Cg-Prkdcscid Il2rgtm1Wjl/SzJ (NSG) mice and followed tumor burden in PIF-treated groups.

## Results

We found that PIF treatment delayed tumor outgrowth in time. A tendency of lower tumor volumes was seen both after administration of immunomodulator peptide and use of additional physical (static magnetic field) treatment. A synergistic effect could be achieved by this combination treatment strategy. Retained or primed expression of glycolipid based tumorassociated antigens on PIF treated melanoma cells could be defined by immunofluorescence FACS and confocal laser microscopy.

## Conclusions

Our results suggest an indirect mechanism of PIF on tumor growth. Retained or enhanced tumor antigen expression is advantageous, as cancerous cells become predisposed to be recognized, while induced activation of antigen presenting cells is beneficial for elimination. Our complementary strategy resulted as effective and provides a new potential modality for cancer control.
